# Both Rerouted and In Situ Biceps Superior Capsular Reconstruction Augmentation During Arthroscopic Rotator Cuff Repair Improve Clinical Outcomes

**DOI:** 10.1002/ars2.70007

**Published:** 2026-05-06

**Authors:** Chang Hee Baek, Bo Taek Kim, Gustavo A. Gil Noriega, Jung Gon Kim, Chaemoon Lim, Paulo J. Llinás Hernández

**Affiliations:** ^1^ Department of Orthopaedic Surgery Yeosu Baek Hospital Jeollanam‐do Republic of Korea; ^2^ Department of Orthopaedic Surgery Valle de Lili Foundation Clinic Cali Colombia

## Abstract

**Purpose:**

To evaluate the clinical and radiologic outcomes at a 2‐year follow‐up of arthroscopic rotator cuff repair (RCR) augmented with superior capsular reconstruction (SCR) using the long head of the biceps tendon (LHBT) in patients with reparable posterosuperior rotator cuff tears.

**Methods:**

This retrospective, multicenter study included patients who underwent arthroscopic RCR with LHBT‐based SCR at 2 institutions: Center A (October 2020 to March 2023) and Center B (June 2022 to July 2024), with a follow‐up of 2 years. Indications included reparable supraspinatus and/or infraspinatus tears with Patte stage 2‐3 retraction and Goutallier grade 3‐4 fatty infiltration. Patients underwent RCR with either in situ LHBT fixation (Center A) or rerouted LHBT fixation to the center of the greater tuberosity (Center B). Exclusion criteria included prior shoulder surgery, infection, irreparable subscapularis tear, or loss to follow‐up. Clinical outcomes were assessed using the visual analog scale, American Shoulder and Elbow Surgeons score, and range of motion. Radiologic evaluation included acromiohumeral distance, Hamada grade, and repair integrity via ultrasound or magnetic resonance imaging.

**Results:**

After excluding 10 patients, 58 were included (N = 26, Center A; N = 32, Center B), and their 2‐year clinical outcomes were compared. Visual analog scale improved from 5.9 ± 1.5 to 2.1 ± 1.3, and American Shoulder and Elbow Surgeons score from 41.2 ± 10.0 to 73.6 ± 12.7 (*P* < .001). Minimal clinically important difference was achieved in 96.5% (visual analog scale) and 94.8% (American Shoulder and Elbow Surgeons). Range of motion improved significantly in forward elevation, abduction, and external rotation. Acromiohumeral distance (increased from 8.1 to 9.0 mm [*P* < .001]) with no progression in Hamada grade. Both centers showed comparable improvements with no significant differences in outcomes between techniques.

**Conclusions:**

Arthroscopic RCR augmented with SCR using the LHBT resulted in significant improvements in pain, function, and range of motion at the 2‐year follow‐up in patients with reparable rotator cuff tears. Both in situ and rerouted LHBT‐based SCR techniques produced comparable clinical and structural outcomes, with no significant differences observed between the 2 methods, with the potential for type II error due to low power.

**Level of Evidences:**

Level III, retrospective comparative case series.

## Graphical abstract

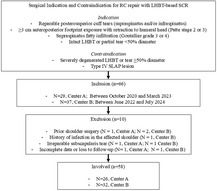

The surgical repair of rotator cuff tears remains a complex challenge, particularly when tendon quality is compromised by early degenerative changes, partial retraction, or subtle muscle atrophy.^1^ Although many of these tears are technically repairable, they carry an increased risk of retear due to the biological and mechanical limitations of the remaining tendon tissue.^2^ To address these concerns and improve the durability of repair, various techniques, such as patch augmentation and marginal convergence, have been introduced to enhance tendon healing and better restore native shoulder biomechanics.^3^, ^4^, ^5^

Superior capsular reconstruction (SCR) was originally developed as a treatment for irrepairable rotator cuff tears to restore superior glenohumeral joint stability.^6^, ^7^ Building on the same biomechanical principles, a modified approach using the long head of the biceps tendon (LHBT) has been proposed.^8^, ^9^, ^10^, ^11^, ^12^ In this technique, the LHBT is preserved at its glenoid origin and fixed to the greater tuberosity, thereby recreating a static superior restraint.^13^, ^14^, ^15^, ^16^, ^17^ This modification partially restores superior stability and reduces superior translation, as shown in a biomechanical study by Denard et al.^13^ supporting its role in augmenting the repaired cuff and preventing superior migration of the humeral head. Compared with traditional graft‐based SCR, this LHBT‐based technique offers several advantages, including shorter operative time, no donor‐site morbidity, reduced cost, and the potential for biologic augmentation due to the presence of viable tenocytes.^10^, ^18^, ^19^ It can be performed in conjunction with standard arthroscopic rotator cuff repair (RCR) and is particularly suited for medium to large tears that are technically repairable but may benefit from both mechanical reinforcement and biological support.^20^, ^21^, ^22^, ^23^, ^24^, ^25^, ^26^

The purpose of this study was to evaluate the clinical and radiologic outcomes at a 2‐year follow‐up of arthroscopic RCR augmented with SCR using LHBT in patients with repairable posterosuperior rotator cuff tears. It was hypothesized that LHBT‐based SCR would yield favorable functional outcomes, with comparable results between the 2 centers.

## METHODS

### Patient Selection

This study was approved by the Institutional Review Board (IRB No. 027‐2018 & P01‐202507‐01‐024). This retrospective multicenter study included patients who underwent arthroscopic RCR augmented with SCR using the LHBT. Patients were enrolled from 2 institutions: Center A (C.H.B; Yeosu Baek Hospital, Yeosu, Republic of Korea) between October 2020 and March 2023, and Center B (P.L; Valle de Lili Foundation Clinic, Cali, Colombia) between June 2022 and July 2024. All procedures were performed by 2 experienced shoulder surgeons at their respective centers. Surgical indications included (1) repairable posterosuperior rotator cuff tears involving the supraspinatus and/or infraspinatus tendons, (2) at least 3 cm of anteroposterior footprint exposure with tendon retraction to the humeral head corresponding to Patte^27^ stage 2 or 3, (3) fatty infiltration of the supraspinatus muscle consistent with Goutallier^28^ grade 3 or 4, and (4) an intact LHBT or a partial LHBT tear involving less than 50% of its diameter. Reparability was assessed intraoperatively by confirming that the torn rotator cuff could be sufficiently mobilized and reattached to fully cover the anatomical footprint without excessive tension. Exclusion criteria were irrepairable rotator cuff tears of the supraspinatus and/or infraspinatus; irrepairable or repairable subscapularis tears deemed nonviable for repair; advanced glenohumeral arthritis (Hamada^29^ grade ≥ 4); prior surgery on the affected shoulder; incomplete clinical or radiologic data; or loss to follow‐up.

### Clinical and Radiologic Assessment

Clinical outcomes were assessed preoperatively and at the 2‐year follow‐up using the Visual Analog Scale (VAS) for pain and the American Shoulder and Elbow Surgeons (ASES) score. Active shoulder range of motion (ROM) was measured with a standardized goniometer, including forward elevation, abduction, and external rotation at the side. Internal rotation was evaluated by identifying the highest vertebral level reached by the thumb behind the back, and scored on a scale from 0 (greater trochanter of the femur) to 10 (T7 vertebra). Radiologic evaluation of glenohumeral arthritis included measurement of the acromiohumeral distance (AHD) and classification according to the Hamada grading system using standard anteroposterior shoulder radiographs. Postoperative integrity of the RCR construct with LHBT‐based SCR was assessed using musculoskeletal ultrasound at Center A and magnetic resonance imaging at Center B during follow‐up.

### Surgical Technique

All procedures were performed arthroscopically under general anesthesia combined with an interscalene nerve block. At Center A (performed by C.H.B), surgeries were conducted with the patient in the lateral decubitus position, while at Center B (performed by P.L), the beach chair position was used. The surgical procedure followed a similar approach at both centers, with minor modifications: Center A performed in situ fixation of the LHBT‐based SCR, while Center B utilized a rerouted technique, securing the LHBT to the center of the greater tuberosity.

Standard arthroscopic portals—anterior, posterior, lateral, and posterolateral—were established. Diagnostic arthroscopy was first performed to evaluate the rotator cuff tear pattern and assess the condition of the LHBT. Reparability of the rotator cuff was confirmed intraoperatively by mobilizing the tendon to cover the anatomical footprint without excessive tension. Subacromial decompression and preparation of the greater tuberosity were carried out using a shaver and burr to create a bleeding bone bed for tendon healing. The LHBT was evaluated for suitability as a graft. In cases where the LHBT showed severe degeneration—defined as involving more than 50% of its diameter—or presented with a type IV SLAP lesion,^30^ it was deemed unsuitable for use in SCR. In such cases, only standard arthroscopic RCR was performed without LHBT‐based SCR.

At Center A, the LHBT was preserved in situ within the bicipital groove and fixed without medialization or centering. A triple‐loaded suture anchor was placed near the lateral edge of the greater tuberosity, just posterior to the LHBT. One suture was looped around the LHBT, and another was passed through it. These sutures were loaded into 2 knotless anchors and inserted anterior to the LHBT: one at the lateral edge of the greater tuberosity and the other near the articular cartilage, effectively compressing the LHBT and completing the LHBT‐based SCR. The remaining suture from the anchor was used to augment the SCR by incorporating it into the RCR via side‐to‐side sutures. An additional 1 or 2 triple‐loaded suture anchors were placed at the center of the greater tuberosity footprint, and RCR was completed using a single‐row or double‐row with a suture‐bridge configuration based on tear characteristics. At Center B, a similar technique was employed, with 1 modification: the LHBT was mobilized posteriorly and fixed at the center of the greater tuberosity rather than in situ. LHBT‐based SCR was performed at this central location, and the RCR was completed using a single‐row or double‐row with suture‐bridge configuration, depending on the tear pattern and tissue quality.

### Postoperative Management

Patients at both centers followed a standardized rehabilitation protocol. Immobilization with an abduction brace was maintained for 4 to 6 weeks, depending on the preoperative characteristics of the rotator cuff tear. Wrist and elbow motion exercises were initiated immediately after surgery. Passive shoulder ROM exercises began at 2 weeks postoperatively, followed by progression to active‐assisted and active exercises at 6 weeks. Strengthening exercises were introduced after 12 weeks. Return to full activities and sports was permitted after 6 months, based on the patient's individual recovery and functional progress.

### Statistical Analysis

Statistical analysis was performed using SPSS version 21 (IBM, Armonk, NY, USA). Continuous variables were analyzed using paired t‐tests or Wilcoxon signed‐rank tests, depending on the normality of the data distribution. Categorical variables were compared using the chi‐square test or Fisher's exact test, as appropriate. The minimal clinically important difference was determined using the 0.5 standard deviation distribution‐based method.^31^ Independent t‐tests were used to compare clinical and radiologic outcomes between the 2 centers. A *P* value of <.05 was considered statistically significant.

## RESULTS

**FIGURE 1:**
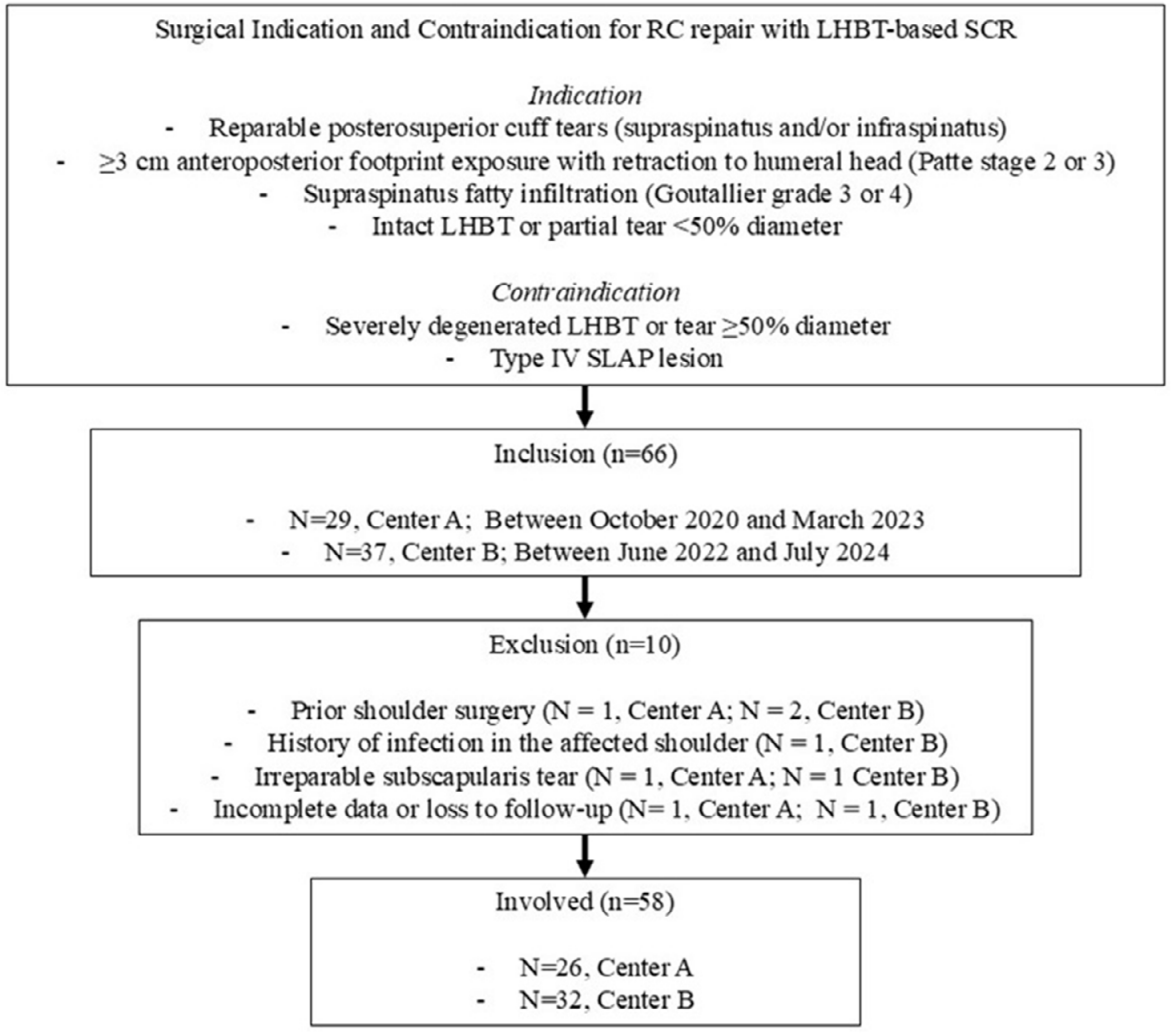
Study enrollment flowchart. (LHBT, long head of biceps tendon; RC, rotator cuff; SCR, superior capsular reconstruction; SLAP, superior labrum from anterior to posterior tear.)

Of the initial 66 patients assessed across the 2 institutions (N = 29, Center A; N = 37, Center B), 8 patients were excluded for the following reasons: prior shoulder surgery (N = 1, Center A; N = 2,Center B), history of infection in the affected shoulder (N = 1, Center B), irreparable subscapularis tear (N = 1, Center A; N = 1, Center B), and incomplete clinical or radiologic data or loss to follow‐up (N = 1, Center A; N = 1, Center B). Thus, a total of 58 patients were included in the final analysis (N = 26, Center A; N = 32, Center B) (Figure 1). The dominant pattern of posterosuperior rotator cuff tears involved isolated supraspinatus tears. Most tears were classified as Patte stage 2 with Goutallier grade 3 fatty infiltration of the supraspinatus. According to the Collin classification, the majority of tears were type D (Table 1).^32^ Although some patients reported anterior biceps pain, those undergoing LHBT‐based SCR all had an intact LHBT or a partial tear <50%, with most complaints related to painful ROM and night pain.

**TABLE 1:** Demographics and Preoperative Rotator Cuff Condition

Variables	Center A (n = 26) In Situ LHBT‐Based SCR	Center B (n = 32) Rerouted LHBT‐Based SCR	*P* Value
Sex, male/female, n (%)	11 (42.3)/15 (57.7)	16 (50.0)	.567
Age (yr), mean ± SD (range)	64.5 ± 6.1 (55‐77)	65.3 ± 8.2 (47‐81)	.700
Dominant arm involvement, n (%)	20 (76.9)	21 (65.6)	.356
Smoking, n (%)	1 (3.8)	0 (0)	.271
Patte stage, n (%)			.691
Stage 2	16 (61.5)	18 (56.2)	
Stage 3	10 (38.4)	14 (43.7)	
Goutallier grade, Mean ± SD (range)			
Subscapularis	1.3 ± 0.7 (0‐2)	1.6 ± 0.9 (0‐3)	.156
Supraspinatus	3.1 ± 0.3 (3‐4)	3.1 ± 0.3 (3‐4)	.792
Infraspinatus	1.8 ± 0.8 (1‐4)	2.1 ± 0.8 (1‐3)	.127
Teres minor	0.4 ± 0.5 (0‐1)	0.5 ± 0.6 (0‐2)	.446
Collin classification, n (%)			.347
Type A	2 (7.7)	0 (0)	
Type B	0 (0)	0 (0)	
Type C	9 (34.6)	13 (40.6)	
Type D	15 (57.7)	19 (59.4)	

LHBT, long head of biceps tendon; SCR, superior capsular reconstruction; SD, standard deviation; yr, year.

For the entire cohort, the VAS pain score significantly improved from 5.9 ± 1.5 preoperatively to 2.1 ± 1.3 at final follow‐up, and ASES score also improved significantly from 41.2 ± 10.0 to 73.6 ± 12.7 (*P* < .001 for both). Regarding the minimal clinically important difference, 96.5% of patients (N = 56) achieved clinically meaningful improvement in VAS scores, and 94.8% (N = 55) in ASES scores. ROM improved significantly postoperatively in forward elevation, abduction, and external rotation at the side, with all *P* < .001. Radiologically, AHD increased significantly from 8.1 ± 2.2 to 9.0 ± 1.9 mm, yet no progression of arthritis was observed, as reflected by unchanged Hamada grades (Table 2).

**TABLE 2:** Clinical Results of Entire Cohort

Variables	Preoperative	Postoperative	*P* Value
Clinical score			
VAS	5.9 ± 1.5	2.1 ± 1.3	<.001∗
ASES	41.2 ± 10.0	73.6 ± 12.7	<.001∗
Active ROM, °			
Forward elevation	119 ± 25	158 ± 22	<.001∗
Abduction	103 ± 24	143 ± 24	<.001∗
External rotation at side	40 ± 10	60 ± 15	<.001∗
Internal rotation at back†	5.3 ± 1.2	5.6 ± 0.8	.388
Radiologic outcome			
AHD, mm	8.1 ± 2.2	9.0 ± 1.9	<.001∗
Hamada grade	1.1 ± 0.3	1.1 ± 0.2	.252

AHD, acromio‐humerale distance; ASES, American Shoulder and Elbow Surgeons score; ROM, range of motion; VAS, visual analog scale.

∗ The significant *P* value is below .05.

† Internal rotation was measured as the level that could be reached by the thumb; 0, greater trochanter; 2, buttock; 4, lumbosacral junction; 6, L3; 8, T12; and 10, T7.

When comparing outcomes between Center A and Center B, both centers showed significant improvements in VAS, ASES scores, and ROM postoperatively. Improvements in all clinical measures, including VAS, ASES scores, ROM measures, or AHD, were comparable between the centers without significant differences. Radiologic outcomes, including postoperative AHD, were also similar between the 2 centers (Table 3). Postoperative integrity of the RCR augmented with LHBT‐based SCR, as assessed by healing and retear rates, showed no significant differences between centers, and the incidence of Popeye deformity was comparable and clinically insignificant at both centers (Table 4).

**TABLE 3:** Comparison of Clinical Results Between the Techniques

Variables	Center A (n = 26) In Situ LHBT‐Based SCR	Center B (n = 32) Rerouted LHBT‐Based SCR	*P* Value
Clinical score			
VAS			
Preoperative	6.1 ± 1.3	6.6 ± 1.5	.218
Postoperative	1.9 ± 1.2	2.2 ± 1.3	.384
*P* value	<.001∗	<.001∗	
ASES			
Preoperative	38.9 ± 7.4	35.6 ± 9.1	.143
Postoperative	71.3 ± 20.0	77.3 ± 9.2	.138
*P* value	<.001∗	<.001∗	
Active ROM, °			
Forward elevation			
Preoperative	119 ± 19	114 ± 28	.446
Postoperative	164 ± 17	170 ± 10	.122
*P* value	<.001∗	<.001∗	
Abduction			
Preoperative	106 ± 23	99 ± 24	.284
Postoperative	147 ± 23	155 ± 12	.097
*P* value	<.001∗	<.001∗	
External rotation at side			
Preoperative	41 ± 6	43 ± 14	.476
Postoperative	64 ± 17	69 ± 11	.207
*P* value	<.001∗	<.001∗	
Internal rotation at back†			
Preoperative	6.3 ± 1.1	6.9 ± 2.5	.282
Postoperative	6.9 ± 1.7	7.3 ± 2.3	.433
*P* value	.124	.380	
Radiologic outcome			
AHD, mm			
Preoperative	7.3 ± 1.4	6.9 ± 1.2	.327
Postoperative	9.0 ± 2.2	9.1 ± 1.8	.816
*P* value	<.001∗	<.001∗	
Hamada grade			
Preoperative	1.1 ± 0.3	1.1 ± 0.3	.824
Postoperative	1.1 ± 0.3	1.0 ± 0.0	.021*
*P* value	.425	.083	

AHD, acromio‐humerale distance; ASES, American Shoulder and Elbow Surgeons score; LHBT, long head of biceps tendon; ROM, range of motion; SCR, superior capsular reconstruction; VAS, visual analog scale.

∗ The significant *P* value is below .05.

† Internal rotation was measured as the level that could be reached by the thumb; 0, greater trochanter; 2, buttock; 4, lumbosacral junction; 6, L3; 8, T12; and 10, T7.

**TABLE 4:** Postoperative Integrity

Variables	Center A (n = 26) In Situ LHBT‐Based SCR	Center B (n = 32) Rerouted LHBT‐Based SCR	*P* Value
Postoperative Popeye sign, n (%)	2 (7.7)	1 (3.1)	.444
Postoperative RC repair with LHBT‐based SCR construct, n (%)			
Intact	18 (69.2)	27 (84.4)	.175
Partial retear	3 (11.5)	3 (9.4)	.792
Retear	5 (19.2)	2 (6.2)	.136

LHBT, long head of biceps tendon; RC, rotator cuff; SCR, superior capsular reconstruction.

## DISCUSSION

The current study showed that arthroscopic RCR augmented with SCR using LHBT resulted in significant improvements in shoulder pain, functional outcomes, and ROM at a minimum 2‐year follow‐up. Notably, no significant clinical or radiologic differences were observed between the 2 surgical techniques: one preserving the LHBT in situ within the bicipital groove (Center A), and the other involving rerouting the LHBT to the center of the greater tuberosity (Center B). Both methods provided reliable biological augmentation to RCR, with high structural integrity and low retear rates. The majority of patients achieved the minimal clinically important difference for both VAS and ASES, confirming meaningful clinical benefit. These findings support the versatility and effectiveness of RCR augmented with LHBT‐based SCR, regardless of the specific method of fixation.

The rationale for utilizing the LHBT in SCR is supported by both biomechanical evidence and anatomical suitability. The superior capsule plays a pivotal role in glenohumeral joint stability by resisting superior translation of the humeral head.^6^, ^33^ Loss of this structure, as seen in large or massive rotator cuff tears, alters joint kinematics and accelerates degenerative changes.^6^, ^17^, ^33^ Ishihara et al.^33^ showed in cadaveric models that superior capsular defects increase multidirectional humeral translation, emphasizing the need for reconstruction. The LHBT, originating from the supraglenoid tubercle and coursing through the bicipital groove, is anatomically well positioned to restore superior capsular integrity. Biomechanical studies have shown that LHBT‐based constructs—whether rerouted or maintained in situ—can effectively reduce superior humeral head migration and restore native joint mechanics.^9^, ^12^, ^13^, ^17^ Additionally, the LHBT provides biologic advantages including viable tenocytes, vascularity, and collagen content, which may enhance tendon‐to‐bone healing.^34^, ^35^, ^36^ Compared with autografts such as fascia lata or dermal allografts, the LHBT eliminates donor‐site morbidity, reduces operative time and cost, and simplifies graft preparation, making it a practical and efficient solution for augmenting RCR.^8^, ^10^, ^18^, ^19^

The clinical outcomes of this study align with a growing body of evidence supporting the use of the LHBT in rotator cuff surgery, particularly in irrepairable or borderline‐repairable cases.^5^, ^11^, ^13^, ^14^, ^16^, ^18^, ^19^, ^21^, ^22^, ^23^, ^24^, ^25^, ^34^, ^37^ Several studies have highlighted its efficacy in reducing pain and improving function, while maintaining low complication rates. Cheppalli et al.,^37^ in a systematic review, reported favorable outcomes using LHBT‐based SCR for irrepairable rotator cuff tears, noting significant improvements in VAS and patient‐reported measures. Similarly, Thamrongskulsiri et al.,^26^ through a meta‐analysis, confirmed consistent benefits from LHBT augmentation in irrepairable rotator cuff tears. Importantly, LHBT‐based SCR has shown promising clinical outcomes not only in irrepairable cases but also when combined with RCR in repairable tears. Kocaoglu et al.^38^ reported superior results in pain relief and functional recovery when partial RCR was combined with LHBT‐based SCR, compared with SCR using a tensor fascia lata autograft alone. Likewise, Llinás et al.^23^ observed reduced retear rates and improved outcomes when LHBT‐based SCR was added to RCR in cases of massive tears. Although the present study focused on repairable tears, the observed improvements in pain, function, and ROM—along with a low retear rate—support the use of LHBT augmentation as a means to enhance healing in this challenging patient population. Nevertheless, these findings must be interpreted in light of contrasting evidence. For example, Jeon et al.^24^ evaluated large anterior L‐shaped tears and found no significant benefit in tendon healing with additional LHBT augmentation compared with partial repair alone, suggesting that not all tear configurations derive equal benefit from augmentation. In some cases, low‐tension, footprint‐preserving repair strategies may be sufficient.

In the current study, no significant differences in clinical outcomes were observed between the in situ and rerouted LHBT techniques, suggesting that the key factor may be the biological contribution of the graft to the superior capsule, rather than the specific trajectory of the tendon. Both in situ and rerouted techniques for SCR using the LHBT have been documented in previous studies, with each showing favorable clinical outcomes.^11^, ^14^, ^16^, ^18^, ^19^, ^21^ Several factors may account for these positive results. As the procedure is performed entirely within the shoulder joint, it reduces the risk of infection and shortens operative time by eliminating the need for graft harvesting or interposition tissue preparation. The LHBT is secured with both medial and lateral anchors, resulting in a tenodesis effect that may provide additional therapeutic benefit in patients with biceps pathology, such as tendinitis or instability. Moreover, this technique may help prevent the progression of cuff tear arthropathy in patients with large to massive rotator cuff tears, likely due to the combined effects of RCR, the downward stabilizing force of the rerouted LHBT, and the space‐occupying nature of the SCR construct.^6^, ^33^ Nevertheless, rerouting the LHBT can lead to increased tension in the tendon, which may cause transient discomfort or bicipital pain during the early postoperative phase.^17^, ^39^ Further study is warranted to clarify the source of postoperative pain in these cases. Overall, these findings underscore the adaptability of the LHBT‐based approach and suggest that the surgical technique can be tailored to the tear configuration, tendon mobility, and intraoperative findings without compromising outcomes.

### Limitations

This study has several limitations. The retrospective, dual‐center design introduces potential variability in patient selection, surgical technique, and imaging follow‐up. Although baseline characteristics were comparable, differences related to the operating surgeons and institutional protocols may have influenced the outcomes. Second, follow‐up imaging was performed using magnetic resonance imaging at Center A and ultrasound at Center B; while both modalities are reliable for assessing tendon integrity, differences in sensitivity may have affected detection of subtle partial retears. Third, there was no a priori power analysis, and there is a significant potential for insufficient power for the primary outcome measure, especially detecting mean differences, which would require a substantially larger sample size to increase power. Consequently, the relatively small cohort size should be considered a limitation, and interpretation of comparative outcomes warrants caution. Fourth, the follow‐up period was limited to 2 years, which may not fully capture the long‐term durability of the RCR with LHBT‐based SCR augmentation. Fifth, this study was multicenter in design, and each center predominantly performed its preferred biceps SCR technique, which may introduce variability and limit direct comparison of the procedures. Lastly, the absence of a control group undergoing standard RCR without augmentation limits the ability to directly attribute improvements to the LHBT‐based SCR technique.

## CONCLUSIONS

Arthroscopic RCR augmented with SCR using the LHBT resulted in significant improvements in pain, function, and ROM at the 2‐year follow‐up in patients with reparable rotator cuff tears. Both in situ and rerouted LHBT‐based SCR techniques produced comparable clinical and structural outcomes, with no significant differences observed between the 2 methods, with the potential for type II error due to low power.

## DISCLOSURES

The authors (C.H.B, B.T.K., G.A.G.N., J.G.K., C.L., P.J.L.H.) declare that they have no known competing financial interests or personal relationships that could have appeared to influence the work reported in this paper.
